# Halogen-bonded zigzag mol­ecular network based upon 1,2-di­iodo­perchloro­benzene and the photoproduct *rctt*-1,3-bis­(pyridin-4-yl)-2,4-di­phenyl­cyclo­butane

**DOI:** 10.1107/S2056989022004200

**Published:** 2022-04-22

**Authors:** Taylor J. Dunning, Eric Bosch, Ryan H. Groeneman

**Affiliations:** a Department of Biological Sciences, Webster University, St. Louis, MO 63119, USA; b Department of Chemistry, Missouri State University, Springfield, MO 65897, USA

**Keywords:** halogen bonding, organic solid state, co-crystal, photoproduct, cyclo­butane, [2 + 2] cyclo­addition reaction

## Abstract

The formation and crystal structure of a zigzag mol­ecular network held together by I⋯N halogen bonds is reported. In particular, the halogen-bond donor is 1,2-di­iodo­perchloro­benzene (**1,2-C_6_I_2_Cl_4_**) while the acceptor is a head-to-tail photoproduct, namely *rctt*-1,3-bis­(pyridin-4-yl)-2,4-di­phenyl­cyclo­butane (***ht*** **-PP**). In this co-crystal (**1,2-C_6_I_2_Cl_4_**)·(***ht*-PP**), the donor acts as a bent two-connected node while the acceptor behaves as a linear linker to form the extended solid. Neighbouring chains pack in a tongue-and-groove-like pattern that engage in various Cl⋯π inter­actions to both the phenyl and pyridyl rings resulting in a supra­molecular two-dimensional sheet.

## Abstract 2

The formation of a halogen-bonded zigzag mol­ecular network based upon 1,2-di­iodo­perchloro­benzene and the head-to-tail photoproduct *rctt* −1,3-bis­(pyridin-4-yl)-2,4-di­phenyl­cyclo­butane is reported. The co-crystal is sustained by I⋯N halogen bonds where the photoproduct acts as a linear linker while the donor behaves as a bent two-connected node within the zigzag chain.

## 1. Chemical context

A continued area of research within crystal engineering is the design and formation of supra­molecular networks that have specific and targeted structures (Yang *et al.*, 2015 ▸; Vantomme & Meijer, 2019 ▸). While the field is diverse and inter­disciplinary, the self-assembly of small mol­ecules to yield purely organic materials continues to be a main focus for materials scientists as well as solid-state chemists (Zhang *et al.*, 2019 ▸). Controlling the overall topology of these assembled supra­molecular networks can easily be achieved by the careful selection of both the node and linker groups typified by metal–organic and supra­molecular coordination frameworks (Jiang *et al.*, 2018 ▸) as well as flexible organic frameworks (Huang *et al.*, 2019 ▸). Halogen bonding continues to be a well-established and reliable non-covalent inter­action in the formation of these supra­molecular networks (Gilday *et al.*, 2015 ▸). A continued goal within our research groups has been the design and construction of halogen-bonded mol­ecular solids containing nodes generated by the [2 + 2] cyclo­addition reaction (Dunning *et al.*, 2021 ▸; Oburn *et al.*, 2020 ▸; Sinnwell *et al.*, 2020 ▸). In each example, the cyclo­butane-based photoproduct accepts I⋯N halogen bonds to form these extended solids. These functionalized photoproducts are ideal components, in the formation of these networks, due to the ability to control the number and position of halogen-bond accepting groups coming off the central cyclo­butane ring (Gan *et al.*, 2018 ▸). Recently, we reported the ability to vary the topology within a pair of halogen-bonded networks by controlling the regiochemistry of the pendant groups (Dunning *et al.*, 2021 ▸). In that contribution, the resulting topology was dictated by the regiochemical position of the 4-pyridyl groups around the cyclo­butane ring. In particular, the incorporation of the head-to-tail photoproduct *rctt*-1,3-bis­(pyridin-4-yl)-2,4-di­phenyl­cyclo­butane (***ht*** **-PP**) or the head-to-head photoproduct *rctt*-1,2-bis­(pyridin-4-yl)-3,4-di­phenyl­cyclo­butane resulted in either a linear or zigzag mol­ecular topology, respectively. In both networks, the halogen-bond donor was 1,4-di­iodo­perchloro­benzene, which acted as a linear linker due to the *para*-position of the two I-atoms. 
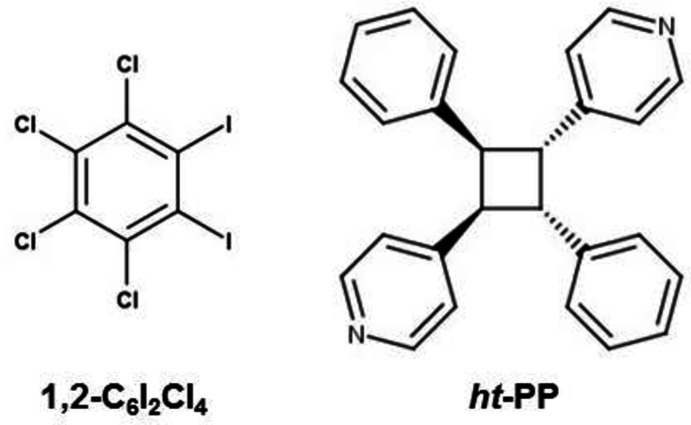


Using this as inspiration, a research project was undertaken to exploit the ability of 1,2-di­iodo­perchloro­benzene (**1,2-C_6_I_2_Cl_4_**) to act as a halogen-bond donor (Bosch *et al.*, 2020 ▸) that would result in a similar zigzag structure when combined with ***ht*** **-PP**, a linear node-based photoproduct. To this end, we report here the synthesis and crystal structure of the co-crystal (**1,2-C_6_I_2_Cl_4_**)·(***ht*** **-PP**) that has a zigzag topology due to the *ortho*-position of the I atoms on the halogen-bond donor. This co-crystal is sustained by I⋯N halogen bonds where neighbouring chains pack in a tongue-and-groove-like pattern. These neighbouring chains engage in various Cl⋯π inter­actions to both the phenyl and pyridyl rings on the photoproduct, resulting in a supra­molecular two-dimensional sheet.

## 2. Structural commentary

**Figure 1:**
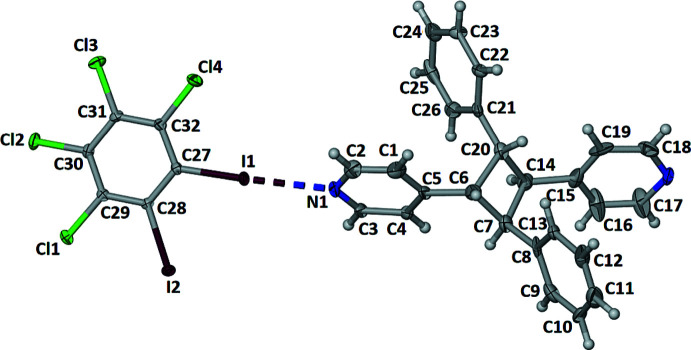
The labelled asymmetric unit of (**1,2-C_6_I_2_Cl_4_**)·(***ht*** **-PP**). Displacement ellipsoids are drawn at the 50% probability level for non-hydrogen atoms while hydrogen atoms are shown as spheres of arbitrary size.

**Figure 2:**
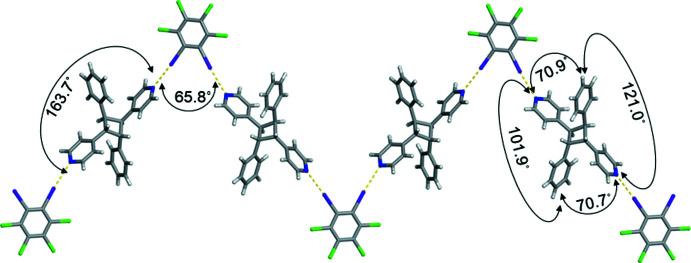
X-ray crystal structure of (**1,2-C_6_I_2_Cl_4_**)·(***ht*** **-PP**) illustrating the zigzag network held together by I⋯N halogen bonds. The determined error in all measured angles is 0.1°. Halogen bonds are represented by yellow dashed lines.

Crystallographic analysis revealed that (**1,2-C_6_I_2_Cl_4_**)·(***ht*** **-PP**) crystallizes in the centrosymmetric monoclinic space group *P*2_1_/*n*. The asymmetric unit contains a full mol­ecule of both **1,2-C_6_I_2_Cl_4_** and ***ht*** **-PP** (Fig. 1 ▸). As a consequence of the *rctt*-stereochemistry within ***ht*** **-PP**, there are two acute [70.7 (1) and 70.9 (1)°] and two obtuse [101.9 (1) and 121.0 (1)°] angles between neighbouring aromatic rings within the photoproduct (Fig. 2 ▸). More important to this contribution, the angle measured between the 4-pyridyl rings and the cyclo­butane has a value of 163.7 (1)°, which allows the photoproduct to act as a linear linker (Fig. 2 ▸). All angles were measured from the centroids of both the aromatic and cyclo­butane rings. As expected, **1,2-C_6_I_2_Cl_4_** engages in two crystallographically unique I⋯N halogen bonds with the 4-pyridyl rings on ***ht*** **-PP** (Fig. 2 ▸). The I1⋯N1 and I2⋯N2^i^ bond distances are 2.809 (6) and 2.927 (6) Å along with bond angles for C27—I1⋯N1 and C28—I2⋯N2^i^ of 177.8 (2) and 175.6 (2)°, respectively [symmetry code: (i) −*x* + 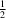 , *y* − 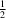 , −*z* + 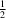 ]. Since the I atoms are in an *ortho*-position, **1,2-C_6_I_2_Cl_4_** acts as a bent halogen-bond donor with a bond angle of 65.8 (1)° measured between the centroid of the donor and the two N atoms (Fig. 2 ▸), forming zigzag chains.

## 3. Supra­molecular features

**Figure 3:**
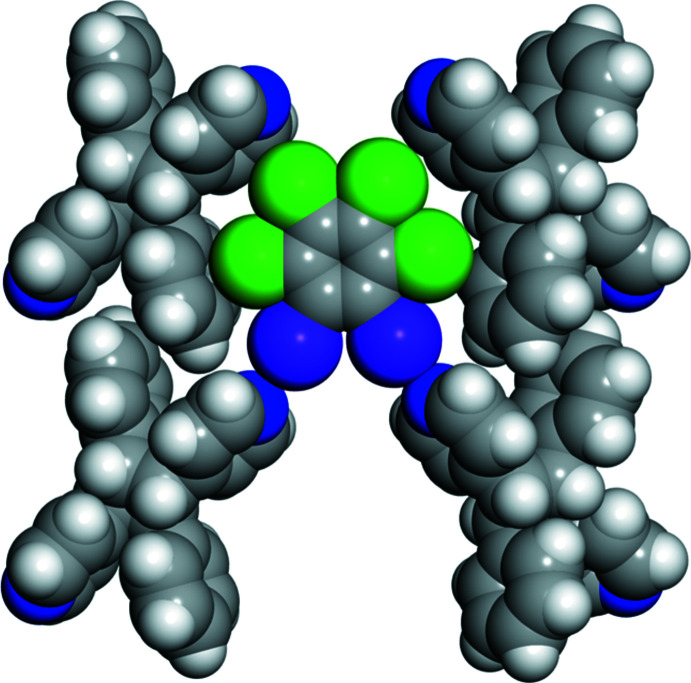
Space-filling model of (**1,2-C_6_I_2_Cl_4_**)·(***ht*** **-PP**) illustrating a closer view of the various Cl⋯π inter­actions.

These zigzag chains inter­act with nearest neighbours by various Cl⋯π inter­actions (Fig. 3 ▸). In particular, all the chlorine atoms on **1,2-C_6_I_2_Cl_4_** are found to inter­act *via* Cl⋯π inter­actions with either 4-pyridyl rings [3.466 (4) and 3.865 (3) Å] or phenyl rings [3.288 (4) and 3.842 (4) Å]. These distances were measured from the chlorine atom to the centroid of the aromatic ring (Youn *et al.*, 2016 ▸). The combination of I⋯N halogen bonds along with the various Cl⋯π inter­actions generates a supra­molecular two-dimensional sheet within (**1,2-C_6_I_2_Cl_4_**)·(***ht*** **-PP**). The polymeric chain is sustained by I⋯N halogen bonds between **1,2-C_6_I_2_Cl_4_** and the photoproduct ***ht*** **-PP**.

**Figure 4:**
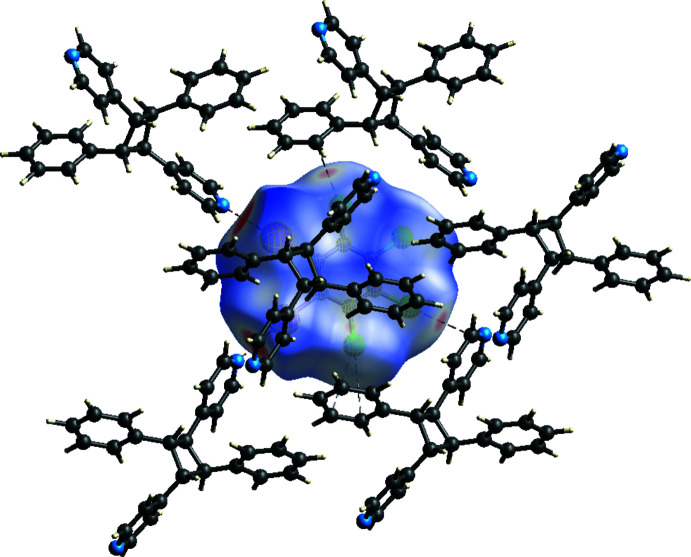
Hirshfeld surface of (**1,2-C_6_I_2_Cl_4_**)·(***ht*** **-PP**) mapped over *d*_norm_ illustrating the I⋯N halogen bonds and Cl⋯π inter­actions.

The various non-covalent inter­actions were also investigated and visualized by using a Hirshfeld surface analysis (Spackman *et al.*, 2021 ▸) mapped over *d*_norm_ (Fig. 4 ▸). The darkest red spots on the surface represent the I⋯N halogen bonds while the lighter red spots are the Cl⋯π inter­actions. The *ortho*-position of the I atoms on the halogen-bond donor makes this mol­ecule behave as a bent two-connecting node, which is required for the formation of a zigzag network.

## 4. Database survey

A search of the Cambridge Crystallographic Database (CSD, Version 5.43, November 2021; Groom *et al.*, 2016 ▸) using *Conquest* (Bruno *et al.*, 2002 ▸) for structures containing **1,2-C_6_I_2_Cl_4_** revealed only one from our earlier study, refcode SUZFUR (Bosch *et al.*, 2020 ▸). A similar search for structures including ***ht*** **-PP** with a halo­benzene that is within the sum of the van der Waals radii of one of the pyridine N atoms yielded two structures, refcodes EQOVUC and EQOWEN (Mondal *et al.*, 2011 ▸). Each of these structures describes a halogen-bonding inter­action within a single mol­ecule, *viz*. 4,4′-(2,4-bis­(4-bromo­phen­yl)cyclo­butane-1,3-di­yl)di­pyridine and 4,4′-(2,4-bis­(4-iodo­phen­yl)cyclo­butane-1,3-di­yl)di­pyridine, respectively.

## 5. Synthesis and crystallization

*Materials and general methods.* The solvents reagent grade ethanol (95%), methyl­ene chloride, and toluene were all purchased from Sigma-Aldrich Chemical (St. Louis, MO, USA) and used as received. In addition, 4,6-di­chloro­resorcinol (**4,6-diCl res**), 4-stilbazole (**SB**), and sodium hydroxide pellets were also purchased from Sigma-Aldrich and were used as received. The [2 + 2] cyclo­addition reaction was conducted in an ACE Glass photochemistry cabinet using UV radiation from a 450 W medium-pressure mercury lamp. The occurrence and yield of the [2 + 2] cyclo­addition reaction was determined by using ^1^H Nuclear Magnetic Resonance Spectroscopy on a Bruker Avance 400 MHz spectrometer with dimethyl sulfoxide (DMSO-*d*_6_) as the solvent. The halogen-bond donor 1,2-di­iodo­perchloro­benzene (**1,2-C_6_I_2_Cl_4_**) was synthesized utilizing a previously published method (Reddy *et al.*, 2006 ▸).

*Synthesis and crystallization.* The formation of the photoreactive co-crystal (**4,6-diCl res**)·(**SB**) was achieved using a previously published approach (Grobelny *et al.*, 2018 ▸). In particular, co-crystals of (**4,6-diCl res**)·(**SB**) were formed by dissolving 50.0 mg of **SB** in 2.0 mL of ethanol, which was then combined with a separate 2.0 mL ethanol solution containing 24.7 mg of **4,6-diCl res** (2:1 molar equivalent). Then the resulting solution was allowed to slowly evaporate. After evaporation of the solvent, the remaining solid was removed and placed between Pyrex glass plates for irradiation. After 20 h of UV exposure, the [2 + 2] cyclo­addition reaction occurred with a 100% yield. The formation of ***ht*** **-PP** was confirmed by ^1^H NMR (Grobelny *et al.*, 2018 ▸) by the complete loss of the olefin peak on **SB** at 7.57 ppm along with the appearance of a cyclo­butane peak at 4.59 ppm (Fig. S1 in the supporting information). The **4,6-diCl res** template was then removed by a base extraction with a 5.0 mL of a 0.2 *M* sodium hydroxide solution that was heated and stirred on a hot plate for 10 minutes. Afterwards, ***ht*** **-PP** was extracted by using three 10 mL aliquots of methyl­ene chloride as the solvent. Then the methyl­ene chloride was removed under vacuum to yield pure ***ht*** **-PP**. The formation of (**1,2-C_6_I_2_Cl_4_**)·(***ht*** **-PP**) was achieved by dissolving 25.0 mg of **1,2-C_6_I_2_Cl_4_** in 2.0 mL of toluene and then combined with a 3.0 mL toluene solution containing 19.4 mg of ***ht*** **-PP** (1:1 molar equivalent). Within two days, single crystals suitable for X-ray diffraction were formed upon loss of some of the solvent by slow evaporation.

## 6. Refinement

Crystal data, data collection and structure refinement details are summarized in Table 1 ▸. Data collection at low temperature, namely 100 K, was facilitated using a Kryoflex system with an accuracy of 1 K. H atoms were included in the refinement at calculated positions.

## Supplementary Material

Crystal structure: contains datablock(s) I. DOI: 10.1107/S2056989022004200/dx2043sup1.cif

Structure factors: contains datablock(s) I. DOI: 10.1107/S2056989022004200/dx2043Isup4.hkl

NMR Data. DOI: 10.1107/S2056989022004200/dx2043sup3.docx

CCDC reference: 2133162

Additional supporting information:  crystallographic information; 3D view; checkCIF report

## Figures and Tables

**Table 1:** Experimental details

Crystal data	
Chemical formula	C_26_H_22_N_2_·C_6_Cl_4_I_2_
*M*_r_	830.11
Crystal system, space group	Monoclinic, *P*2_1_/*n*
Temperature (K)	100
*a*, *b*, *c* (Å)	9.6519 (6), 28.3120 (16), 11.1909 (6)
β (°)	92.154 (1)
*V* (Å^3^)	3055.9 (3)
*Z*	4
Radiation type	Mo *K*α
μ (mm^−1^)	2.43
Crystal size (mm)	0.55 × 0.23 × 0.17

Data collection	
Diffractometer	Bruker APEXII CCD
Absorption correction	Multi-scan (*SADABS*; Bruker, 2014 ▸)
*T*_min_, *T*_max_	0.690, 0.746
No. of measured, independent and observed [*I* > 2σ(*I*)] reflections	39572, 6730, 6601
*R*_int_	0.024
(sin θ/λ)_max_ (Å^−1^)	0.641

Refinement	
*R*[*F* ^2^ > 2σ(*F* ^2^)], *wR*(*F* ^2^), *S*	0.056, 0.118, 1.39
No. of reflections	6730
No. of parameters	361
H-atom treatment	H-atom parameters constrained
Δρ_max_, Δρ_min_ (e Å^−3^)	1.85, −1.11

Computer programs: *SMART* and *SAINT* (Bruker, 2014 ▸), *SHELXT2018/2* (Sheldrick, 2015*a* ▸), *SHELXL2018/3* (Sheldrick, 2015*b* ▸) and *X-SEED* (Barbour, 2020 ▸).

## supplementary crystallographic information

### Crystal data

**Table:**

C_26_H_22_N_2_·C_6_Cl_4_I_2_	*F*(000) = 1608
*M**_r_* = 830.11	*D*_x_ = 1.804 Mg m^−^^3^
Monoclinic, *P*2_1_/*n*	Mo *K*α radiation, λ = 0.71073 Å
*a* = 9.6519 (6) Å	Cell parameters from 9788 reflections
*b* = 28.3120 (16) Å	θ = 2.2–27.1°
*c* = 11.1909 (6) Å	µ = 2.43 mm^−^^1^
β = 92.154 (1)°	*T* = 100 K
*V* = 3055.9 (3) Å^3^	Cut block, gold
*Z* = 4	0.55 × 0.23 × 0.17 mm

### Data collection

**Table:**

Bruker APEXII CCD diffractometer	6730 independent reflections
Radiation source: fine-focus sealed tube	6601 reflections with *I* > 2σ(*I*)
Graphite monochromator	*R*_int_ = 0.024
Detector resolution: 8.3660 pixels mm^-1^	θ_max_ = 27.1°, θ_min_ = 1.4°
phi and ω scans	*h* = −12→12
Absorption correction: multi-scan (SADABS; Bruker, 2014)	*k* = −36→36
*T*_min_ = 0.690, *T*_max_ = 0.746	*l* = −14→14
39572 measured reflections	

### Refinement

**Table:**

Refinement on *F*^2^	Primary atom site location: dual
Least-squares matrix: full	Hydrogen site location: inferred from neighbouring sites
*R*[*F*^2^ > 2σ(*F*^2^)] = 0.056	H-atom parameters constrained
*wR*(*F*^2^) = 0.118	*w* = 1/[σ^2^(*F*_o_^2^) + 34.6345*P*] where *P* = (*F*_o_^2^ + 2*F*_c_^2^)/3
*S* = 1.39	(Δ/σ)_max_ = 0.001
6730 reflections	Δρ_max_ = 1.85 e Å^−^^3^
361 parameters	Δρ_min_ = −1.11 e Å^−^^3^
0 restraints	

### Special details

**Table:**

Geometry. All esds (except the esd in the dihedral angle between two l.s. planes) are estimated using the full covariance matrix. The cell esds are taken into account individually in the estimation of esds in distances, angles and torsion angles; correlations between esds in cell parameters are only used when they are defined by crystal symmetry. An approximate (isotropic) treatment of cell esds is used for estimating esds involving l.s. planes.

### Fractional atomic coordinates and isotropic or equivalent isotropic displacement parameters (Å^2^)

**Table:**

	*x*	*y*	*z*	*U*_iso_*/*U*_eq_	
I1	0.42402 (5)	0.67570 (2)	0.76670 (4)	0.01997 (11)	
Cl1	0.32159 (16)	0.50027 (5)	1.01755 (15)	0.0205 (3)	
N1	0.4404 (7)	0.7347 (2)	0.5659 (5)	0.0289 (14)	
C1	0.5615 (9)	0.7937 (3)	0.4624 (7)	0.0366 (19)	
H1	0.643186	0.812241	0.457280	0.044*	
I2	0.33844 (4)	0.55063 (2)	0.75026 (4)	0.01986 (11)	
Cl2	0.36316 (19)	0.54909 (6)	1.26078 (14)	0.0278 (4)	
N2	0.2232 (7)	0.9965 (2)	−0.0317 (5)	0.0284 (14)	
C2	0.5502 (8)	0.7620 (3)	0.5512 (7)	0.0346 (18)	
H2	0.626199	0.758897	0.607178	0.041*	
Cl3	0.45076 (19)	0.65490 (7)	1.27684 (15)	0.0277 (4)	
C3	0.3349 (8)	0.7400 (2)	0.4888 (6)	0.0265 (15)	
H3	0.255056	0.720864	0.497785	0.032*	
Cl4	0.4745 (2)	0.71377 (6)	1.04655 (16)	0.0308 (4)	
C4	0.3349 (8)	0.7723 (2)	0.3944 (6)	0.0291 (16)	
H4	0.255937	0.775788	0.341925	0.035*	
C5	0.4541 (9)	0.7993 (2)	0.3794 (6)	0.0290 (16)	
C6	0.4776 (8)	0.8351 (3)	0.2821 (7)	0.0307 (16)	
H6	0.575422	0.833443	0.255902	0.037*	
C7	0.3761 (9)	0.8370 (3)	0.1726 (7)	0.0319 (17)	
H7	0.307057	0.810670	0.173423	0.038*	
C8	0.4407 (9)	0.8410 (3)	0.0559 (7)	0.0328 (18)	
C9	0.3756 (10)	0.8186 (3)	−0.0507 (9)	0.041 (2)	
H9	0.289941	0.802314	−0.045522	0.049*	
C10	0.4405 (12)	0.8214 (3)	−0.1583 (7)	0.045 (2)	
H10	0.398415	0.806288	−0.226264	0.054*	
C11	0.5575 (12)	0.8439 (3)	−0.1709 (9)	0.052 (3)	
H11	0.597664	0.845191	−0.247032	0.062*	
C12	0.6206 (11)	0.8650 (3)	−0.0772 (8)	0.045 (2)	
H12	0.704995	0.881530	−0.087581	0.054*	
C13	0.5657 (8)	0.8634 (2)	0.0336 (7)	0.0286 (16)	
H13	0.615099	0.878176	0.098356	0.034*	
C14	0.3151 (8)	0.8844 (3)	0.2236 (7)	0.0317 (16)	
H14	0.229183	0.877278	0.267459	0.038*	
C15	0.2863 (9)	0.9255 (3)	0.1365 (7)	0.0345 (19)	
C16	0.1784 (10)	0.9223 (4)	0.0598 (11)	0.063 (3)	
H16	0.120838	0.895069	0.060582	0.075*	
C17	0.1494 (10)	0.9574 (4)	−0.0196 (11)	0.062 (3)	
H17	0.069486	0.953656	−0.070986	0.074*	
C18	0.3302 (10)	1.0006 (3)	0.0436 (8)	0.042 (2)	
H18	0.386524	1.028064	0.040173	0.050*	
C19	0.3647 (10)	0.9651 (4)	0.1307 (8)	0.050 (3)	
H19	0.442388	0.969243	0.184286	0.060*	
C20	0.4378 (7)	0.8881 (3)	0.3126 (7)	0.0258 (15)	
H20	0.508646	0.909802	0.279637	0.031*	
C21	0.4104 (9)	0.9027 (2)	0.4437 (7)	0.0294 (16)	
C22	0.5038 (8)	0.9328 (2)	0.5010 (7)	0.0258 (15)	
H22	0.578466	0.944934	0.457292	0.031*	
C23	0.4934 (7)	0.9460 (3)	0.6190 (6)	0.0259 (15)	
H23	0.560861	0.966418	0.655345	0.031*	
C24	0.3846 (8)	0.9296 (3)	0.6843 (8)	0.0337 (17)	
H24	0.375429	0.938316	0.765629	0.040*	
C25	0.2873 (8)	0.8993 (3)	0.6255 (9)	0.040 (2)	
H25	0.210484	0.887897	0.667556	0.047*	
C26	0.3022 (9)	0.8863 (3)	0.5083 (8)	0.0340 (17)	
H26	0.236364	0.865432	0.471384	0.041*	
C27	0.4096 (6)	0.6337 (2)	0.9214 (6)	0.0165 (12)	
C28	0.3769 (6)	0.5853 (2)	0.9156 (5)	0.0142 (11)	
C29	0.3655 (6)	0.5593 (2)	1.0213 (6)	0.0162 (12)	
C30	0.3846 (6)	0.5812 (2)	1.1325 (6)	0.0172 (12)	
C31	0.4211 (6)	0.6286 (2)	1.1393 (5)	0.0168 (12)	
C32	0.4323 (7)	0.6547 (2)	1.0351 (6)	0.0190 (13)	

### Atomic displacement parameters (Å^2^)

**Table:**

	*U*^11^	*U*^22^	*U*^33^	*U*^12^	*U*^13^	*U*^23^
I1	0.0268 (2)	0.01597 (19)	0.0172 (2)	0.00231 (16)	0.00253 (15)	0.00356 (15)
Cl1	0.0182 (7)	0.0156 (7)	0.0277 (8)	−0.0016 (6)	0.0019 (6)	0.0031 (6)
N1	0.046 (4)	0.017 (3)	0.024 (3)	0.005 (3)	0.001 (3)	0.006 (2)
C1	0.038 (4)	0.042 (5)	0.030 (4)	−0.015 (4)	0.011 (3)	−0.003 (3)
I2	0.0240 (2)	0.0190 (2)	0.0166 (2)	−0.00125 (16)	0.00060 (15)	−0.00368 (15)
Cl2	0.0359 (9)	0.0298 (9)	0.0178 (7)	0.0041 (7)	0.0029 (7)	0.0089 (6)
N2	0.039 (4)	0.029 (3)	0.017 (3)	0.012 (3)	0.004 (3)	0.008 (2)
C2	0.030 (4)	0.038 (4)	0.036 (4)	0.008 (3)	−0.001 (3)	0.000 (3)
Cl3	0.0316 (9)	0.0345 (9)	0.0170 (7)	−0.0033 (7)	0.0026 (6)	−0.0080 (7)
C3	0.037 (4)	0.021 (3)	0.022 (3)	−0.011 (3)	0.005 (3)	0.003 (3)
Cl4	0.0479 (11)	0.0180 (8)	0.0268 (8)	−0.0092 (7)	0.0075 (8)	−0.0041 (6)
C4	0.042 (4)	0.020 (3)	0.024 (4)	0.003 (3)	−0.012 (3)	0.001 (3)
C5	0.050 (5)	0.018 (3)	0.019 (3)	−0.006 (3)	0.007 (3)	0.001 (3)
C6	0.032 (4)	0.030 (4)	0.030 (4)	0.002 (3)	−0.002 (3)	−0.002 (3)
C7	0.040 (4)	0.027 (4)	0.028 (4)	−0.002 (3)	0.003 (3)	0.007 (3)
C8	0.042 (4)	0.025 (4)	0.032 (4)	0.018 (3)	0.018 (3)	0.019 (3)
C9	0.047 (5)	0.021 (4)	0.055 (5)	−0.002 (3)	0.004 (4)	0.008 (4)
C10	0.086 (8)	0.032 (4)	0.016 (3)	0.023 (5)	−0.004 (4)	−0.002 (3)
C11	0.081 (8)	0.038 (5)	0.038 (5)	0.025 (5)	0.015 (5)	0.005 (4)
C12	0.063 (6)	0.040 (5)	0.034 (5)	0.022 (4)	0.029 (4)	0.013 (4)
C13	0.041 (4)	0.020 (3)	0.025 (4)	−0.002 (3)	0.001 (3)	0.006 (3)
C14	0.034 (4)	0.036 (4)	0.025 (4)	−0.003 (3)	0.005 (3)	0.003 (3)
C15	0.040 (4)	0.043 (5)	0.021 (3)	0.022 (4)	0.010 (3)	0.004 (3)
C16	0.032 (5)	0.058 (6)	0.098 (9)	0.005 (4)	0.001 (5)	0.047 (6)
C17	0.034 (5)	0.055 (6)	0.095 (9)	−0.003 (4)	−0.028 (5)	0.035 (6)
C18	0.046 (5)	0.026 (4)	0.052 (5)	0.008 (4)	−0.003 (4)	−0.012 (4)
C19	0.052 (6)	0.059 (6)	0.036 (5)	0.032 (5)	−0.026 (4)	−0.029 (4)
C20	0.020 (3)	0.025 (4)	0.032 (4)	0.002 (3)	0.002 (3)	0.005 (3)
C21	0.044 (4)	0.016 (3)	0.028 (4)	−0.001 (3)	−0.011 (3)	0.007 (3)
C22	0.025 (3)	0.020 (3)	0.032 (4)	0.002 (3)	−0.007 (3)	−0.004 (3)
C23	0.019 (3)	0.029 (4)	0.029 (4)	−0.001 (3)	0.000 (3)	−0.008 (3)
C24	0.035 (4)	0.026 (4)	0.041 (4)	0.009 (3)	0.008 (3)	0.002 (3)
C25	0.019 (4)	0.025 (4)	0.076 (6)	0.005 (3)	0.011 (4)	0.021 (4)
C26	0.032 (4)	0.029 (4)	0.039 (4)	0.002 (3)	−0.011 (3)	−0.001 (3)
C27	0.015 (3)	0.015 (3)	0.019 (3)	0.002 (2)	0.000 (2)	0.002 (2)
C28	0.010 (3)	0.018 (3)	0.015 (3)	0.003 (2)	0.000 (2)	−0.002 (2)
C29	0.011 (3)	0.017 (3)	0.021 (3)	−0.001 (2)	−0.001 (2)	0.000 (2)
C30	0.013 (3)	0.021 (3)	0.018 (3)	0.004 (2)	0.002 (2)	0.003 (2)
C31	0.014 (3)	0.023 (3)	0.014 (3)	0.000 (2)	−0.001 (2)	−0.003 (2)
C32	0.022 (3)	0.016 (3)	0.019 (3)	−0.002 (2)	0.002 (2)	0.000 (2)

### Geometric parameters (Å, º)

**Table:**

I1—C27	2.110 (6)	C12—C13	1.367 (11)
Cl1—C29	1.725 (6)	C12—H12	0.9500
N1—C3	1.318 (10)	C13—H13	0.9500
N1—C2	1.327 (11)	C14—C20	1.522 (10)
C1—C2	1.348 (11)	C14—C15	1.535 (11)
C1—C5	1.375 (12)	C14—H14	1.0000
C1—H1	0.9500	C15—C16	1.328 (14)
I2—C28	2.115 (6)	C15—C19	1.356 (14)
Cl2—C30	1.718 (6)	C16—C17	1.356 (13)
N2—C18	1.314 (11)	C16—H16	0.9500
N2—C17	1.327 (12)	C17—H17	0.9500
C2—H2	0.9500	C18—C19	1.431 (13)
Cl3—C31	1.725 (6)	C18—H18	0.9500
C3—C4	1.397 (10)	C19—H19	0.9500
C3—H3	0.9500	C20—C21	1.556 (11)
Cl4—C32	1.725 (7)	C20—H20	1.0000
C4—C5	1.397 (11)	C21—C26	1.375 (12)
C4—H4	0.9500	C21—C22	1.381 (10)
C5—C6	1.513 (10)	C22—C23	1.381 (10)
C6—C7	1.541 (11)	C22—H22	0.9500
C6—C20	1.588 (10)	C23—C24	1.382 (11)
C6—H6	1.0000	C23—H23	0.9500
C7—C8	1.472 (10)	C24—C25	1.415 (12)
C7—C14	1.583 (11)	C24—H24	0.9500
C7—H7	1.0000	C25—C26	1.375 (13)
C8—C13	1.393 (11)	C25—H25	0.9500
C8—C9	1.472 (13)	C26—H26	0.9500
C9—C10	1.381 (13)	C27—C28	1.406 (9)
C9—H9	0.9500	C27—C32	1.414 (9)
C10—C11	1.309 (15)	C28—C29	1.400 (9)
C10—H10	0.9500	C29—C30	1.396 (9)
C11—C12	1.335 (15)	C30—C31	1.389 (9)
C11—H11	0.9500	C31—C32	1.389 (9)
			
C3—N1—C2	117.0 (6)	C19—C15—C14	124.7 (8)
C2—C1—C5	119.6 (8)	C15—C16—C17	120.8 (10)
C2—C1—H1	120.2	C15—C16—H16	119.6
C5—C1—H1	120.2	C17—C16—H16	119.6
C18—N2—C17	114.8 (7)	N2—C17—C16	125.5 (9)
N1—C2—C1	124.5 (8)	N2—C17—H17	117.2
N1—C2—H2	117.7	C16—C17—H17	117.2
C1—C2—H2	117.7	N2—C18—C19	122.2 (9)
N1—C3—C4	123.2 (7)	N2—C18—H18	118.9
N1—C3—H3	118.4	C19—C18—H18	118.9
C4—C3—H3	118.4	C15—C19—C18	119.9 (8)
C5—C4—C3	118.2 (7)	C15—C19—H19	120.1
C5—C4—H4	120.9	C18—C19—H19	120.1
C3—C4—H4	120.9	C14—C20—C21	118.6 (6)
C1—C5—C4	117.4 (7)	C14—C20—C6	89.1 (6)
C1—C5—C6	115.7 (7)	C21—C20—C6	120.3 (6)
C4—C5—C6	126.9 (7)	C14—C20—H20	109.1
C5—C6—C7	119.1 (7)	C21—C20—H20	109.1
C5—C6—C20	115.8 (6)	C6—C20—H20	109.1
C7—C6—C20	89.3 (6)	C26—C21—C22	117.4 (7)
C5—C6—H6	110.3	C26—C21—C20	124.4 (7)
C7—C6—H6	110.3	C22—C21—C20	118.1 (7)
C20—C6—H6	110.3	C23—C22—C21	122.8 (7)
C8—C7—C6	115.5 (7)	C23—C22—H22	118.6
C8—C7—C14	115.4 (6)	C21—C22—H22	118.6
C6—C7—C14	88.6 (6)	C22—C23—C24	119.9 (7)
C8—C7—H7	111.8	C22—C23—H23	120.0
C6—C7—H7	111.8	C24—C23—H23	120.0
C14—C7—H7	111.8	C23—C24—C25	117.5 (8)
C13—C8—C7	126.4 (8)	C23—C24—H24	121.3
C13—C8—C9	113.4 (7)	C25—C24—H24	121.3
C7—C8—C9	120.2 (8)	C26—C25—C24	121.0 (8)
C10—C9—C8	119.2 (8)	C26—C25—H25	119.5
C10—C9—H9	120.4	C24—C25—H25	119.5
C8—C9—H9	120.4	C21—C26—C25	121.3 (7)
C11—C10—C9	122.9 (9)	C21—C26—H26	119.3
C11—C10—H10	118.5	C25—C26—H26	119.3
C9—C10—H10	118.5	C28—C27—C32	118.6 (6)
C10—C11—C12	120.3 (9)	C28—C27—I1	122.2 (5)
C10—C11—H11	119.9	C32—C27—I1	119.2 (5)
C12—C11—H11	119.9	C29—C28—C27	119.8 (6)
C11—C12—C13	121.1 (10)	C29—C28—I2	118.6 (4)
C11—C12—H12	119.5	C27—C28—I2	121.6 (4)
C13—C12—H12	119.5	C30—C29—C28	120.6 (6)
C12—C13—C8	123.1 (8)	C30—C29—Cl1	118.4 (5)
C12—C13—H13	118.4	C28—C29—Cl1	121.0 (5)
C8—C13—H13	118.4	C31—C30—C29	120.1 (6)
C20—C14—C15	118.8 (7)	C31—C30—Cl2	120.3 (5)
C20—C14—C7	90.1 (6)	C29—C30—Cl2	119.6 (5)
C15—C14—C7	118.3 (6)	C30—C31—C32	119.8 (6)
C20—C14—H14	109.4	C30—C31—Cl3	120.0 (5)
C15—C14—H14	109.4	C32—C31—Cl3	120.2 (5)
C7—C14—H14	109.4	C31—C32—C27	121.1 (6)
C16—C15—C19	116.8 (8)	C31—C32—Cl4	118.7 (5)
C16—C15—C14	118.5 (9)	C27—C32—Cl4	120.2 (5)

### Hydrogen-bond geometry (Å, º)

**Table:**

*D*—H···*A*	*D*—H	H···*A*	*D*···*A*	*D*—H···*A*
C17—H17···Cl2^i^	0.95	2.69	3.632 (10)	172

Symmetry code: (i) *x*−1/2, −*y*+3/2, *z*−3/2.
