# Supplementary material for: Halogen-bonded zigzag mol­ecular network based upon 1,2-di­iodo­perchloro­benzene and the photoproduct rctt-1,3-bis­(pyridin-4-yl)-2,4-di­phenyl­cyclo­butane
Source: Acta Crystallogr E Crystallogr Commun. 2022 Apr 22;78(Pt 5):506–9. doi: 10.1107/S2056989022004200 (PMC9069512; doi:10.1107/S2056989022004200)
Supplement: Supplementary file 3 [file e-78-00506-sup3.docx]

Supporting information

b

a

a

b

**Figure S1: ^1^**H NMR spectrum of (**4,6-diCl res**)•(***ht*-PP**) after 20 hours of irradiation of UV resulting in a quantitative [2 + 2] cycloaddition reaction (400 MHz, DMSO-*d*_6_).
